# Hepatitis Awareness Month and National Hepatitis Testing Day — May 2015

**Published:** 2015-05-08

This month marks the 20th anniversary of Hepatitis Awareness Month and the 4th National Hepatitis Testing Day (May 19) in the United States. Although care and treatment can be life-saving, many of the 3 million persons estimated to be living with hepatitis C virus (HCV) infection are unaware of their infection and are not receiving preventive services and medical management. In addition, an emerging epidemic of HCV infection among a new demographic of persons who inject drugs is unfolding in several areas throughout the nation. Guided by the goals of the 2014 U.S. Department of Health and Human Services *Action Plan for the Prevention, Care, and Treatment of Viral Hepatitis* (1), CDC continues its activities to expand access to HCV testing, care, and treatment to stem morbidity and mortality, and to reduce HCV infections caused by drug use behaviors. Efforts to address each of these strategic imperatives are highlighted by the two reports in this issue of *MMWR*.

The first report shows that trends in new cases of HCV infection are highly correlated with trends in substance abuse treatment admissions for opioid dependency and opioid injection in four states in the central Appalachian Region. The second report describes strategies for integrating HCV testing into primary care settings. These reports demonstrate how data can be used to identify patterns of risk for HCV transmission among persons who inject drugs and how programs can be successfully implemented to identify persons disproportionately affected by HCV infection and ensure they receive appropriate medical care and treatment.
